# RESIDENTIAL SEGREGATION AND DEPRESSIVE SYMPTOMS IN OLDER CHINESE IMMIGRANTS: MEDIATING ROLE OF SOCIAL PROCESSES

**DOI:** 10.1093/geroni/igad104.1410

**Published:** 2023-12-21

**Authors:** Ke Li, Fengyan Tang, Yanping Jiang, Andrea Rosso

**Affiliations:** University of New Hampshire, Durham, New Hampshire, United States; University of Pittsburgh, Pittsburgh, Pennsylvania, United States; Rutgers University, New Brunswick, New Jersey, United States; University of Pittsburgh, Pittsburgh, Pennsylvania, United States

## Abstract

Older Chinese immigrants are particularly at risk for depression due to acculturative stress and language barriers. Residential segregation with respect to language use may play an important role in mental health of historically marginalized immigrant populations. Previous research provided mixed evidence about the segregation effect among older Latino and Asian immigrants. Guided by a model of social processes, we examined the direct and indirect effects of residential segregation on depressive symptoms via multiple mechanisms of acculturation, discrimination, social network, social support, social strain, and social engagement. Four waves of depressive symptoms were assessed in the Population Study of Chinese Elderly (2011-2019, N=1,970), and linked to the 2010-2014 American Community Survey estimates of neighborhood context. Residential segregation was measured by the Index of Concentrations at the Extremes that simultaneously assesses Chinese and English language use within a given census tract. Latent growth curve models with adjusted cluster robust standard errors were estimated after controlling for individual-level factors. Residents of segregated Chinese-speaking neighborhoods had fewer baseline depressive symptoms but a slower decline rate than those living in segregated toward English-only speaking neighborhoods. Racial discrimination, social strain, and social engagement partially mediated the association between segregation and initial level; social strain and social engagement also partially mediated the association with decline in depressive symptoms. This study demonstrates the importance of residential segregation and social processes in shaping mental well-being among older Chinese immigrants and suggests potential mechanisms to alleviate mental health risks.

